# Social well-being is associated with slower multisystem aging estimated from a single brain MRI scan: prospective evidence from MIDUS

**DOI:** 10.21203/rs.3.rs-11292832/v1

**Published:** 2026-10-11

**Authors:** Minwoo Lee, Sara M. Garza-Gonzalez, Stacey M. Schaefer, Kyle Bourassa, Casey K. Brown

**Affiliations:** Georgetown University; Georgetown University; University of Wisconsin–Madison; Georgetown University; Georgetown University

**Keywords:** well-being, pace of aging, biomarker, DunedinPACNI, volunteering

## Abstract

Subjective well-being is increasingly recognized as critical for health across the adult lifespan. Yet, it is unclear whether separable dimensions of well-being are differentially associated with biological aging. Using data from the Midlife in the United States (MIDUS) study (*N* = 213; age = 56.2 ± 13.6), we modeled the multifaceted structure of subjective well-being (i.e., private/eudaimonic, social, and hedonic well-being) and examined its prospective association with DunedinPACNI, a novel brain MRI-derived biomarker trained to estimate the pace of biological aging. We found that the social dimension of well-being was consistently associated with a slower pace of aging after sequentially controlling for demographic, health, lifestyle, and social covariates. Associations with private/eudaimonic well-being and hedonic well-being were rendered non-significant once health and lifestyle factors were accounted for. Data-driven variable selection further identified social well-being as the most stable predictor of DunedinPACNI among the well-being dimensions. Lastly, using community volunteering as an illustrative example of a modifiable, health-promoting behavior, we found an indirect association with a slower pace of aging via social well-being that was not detected when well-being was modeled as a broader construct. These findings suggest that a fine-grained characterization of subjective well-being may yield novel insights into the psychosocial mechanisms supporting healthy aging. They also provide the first evidence that psychosocial correlates of the pace of aging can be detected from a single structural MRI scan, which may extend the evidence base for aging research to cohorts that lack biospecimens.

## Introduction

Human populations are aging worldwide. This demographic shift comes with a rising burden of age-related and neurodegenerative diseases^1^. Identifying targets for scalable, preventive interventions has become a public health priority, especially given that many health conditions in late life remain difficult to treat once they develop^2^. Psychosocial factors are increasingly recognized as an important target for prevention^3^, as they can be more readily modified through individual- and community-level interventions^4^ compared to many structural determinants of health, such as socioeconomic inequalities and environmental exposures^5,6^.

Subjective well-being is one such psychosocial factor that holds promise. Broadly referring to the cognitive and affective evaluations people make of their lives, subjective well-being characterizes the sense that one is flourishing as an individual and connected to a supportive community^7–9^. As an integrative appraisal across multiple life domains, subjective well-being has been shown to predict a wide range of positive health outcomes, including lower systemic inflammation, reduced risk of chronic disease, and greater longevity^10–13^.

More recently, studies have also linked subjective well-being to the “pace of aging,” or the rate of physiological and functional decline across multiple organ systems^14^. Pace-of-aging measures represent a newer generation of aging biomarkers. Unlike traditional age-prediction algorithms that are trained on cross-sectional samples and estimate an “age gap” (whether someone is estimated to be older or younger than their chronological age),^15^ pace-of-aging measures are instead trained on longitudinal, within-person changes across multiple health indicators and directly estimate the rate of biological aging^14^. This approach is less susceptible to biases associated with cross-sectional age prediction and may better predict longitudinal health outcomes^16,17^. Studies to date have found that high subjective well-being is associated with a slower pace of aging^18,19^, positioning it as a candidate “resilience factor” for healthier aging trajectories^12^.

Two limitations, however, constrain the generalizability and translational potential of these findings. First, many studies in the aging literature have relied on a narrow operationalization of well-being, often using a single instrument to assess constructs such as positive affect or purpose in life^18,20^. Psychological theories instead distinguish multiple dimensions of well-being^21^. Hedonic well-being encompasses global life satisfaction and the balance of positive and negative affect^22^. Eudaimonic well-being, in contrast, reflects optimal functioning and can be further differentiated into private (e.g., sense of purpose, growth, autonomy) and social components (e.g., perceived integration, contribution, positive relationships)^7,23^. Although closely related, these dimensions are statistically separable^24^, show partly distinct cognitive and neural substrates^25^, and differ in their associations with health outcomes^26^. Modeling well-being as a unitary construct thus risks conflating dimensions that relate to aging through different pathways, obscuring which dimensions represent the most promising targets for intervention. Second, associations between subjective well-being and a slower pace of aging have been demonstrated only using blood-derived DNA methylation measures^18^. Whether this association extends to pace of aging estimates derived from other biological modalities, such as brain images, remains unknown. This is important because different biological measures may capture partly distinct aspects of the aging process^27^.

This study aims to narrow both gaps. We characterize subjective well-being as a set of partially separable dimensions and assess their prospective associations with DunedinPACNI, a novel whole-body pace of biological aging measure calculated using a single brain magnetic resonance imaging (MRI) scan^28^. We draw on the Midlife in the United States (MIDUS) study^29^, which offers rich multimodal data from nationally sampled, community-dwelling adults. We hypothesized that these well-being dimensions would show distinct patterns of association with DunedinPACNI. Identifying such patterns could sharpen our understanding of the aspects of well-being that are most relevant to the pace of aging and help identify more specific targets for intervention. We conclude by illustrating the translational utility of this approach using community volunteering, a prosocial behavior widely implicated in healthy aging. Specifically, we tested whether volunteering was indirectly associated with DunedinPACNI through particular dimensions of subjective well-being and whether these pathways could reliably be identified under broader characterizations of well-being.

## Materials and Methods

### Participant characteristics

Data were drawn from MIDUS 3 (M3) and MIDUS Refresher 1 (MR1). M3 was conducted between 2013 and 2022 as a second longitudinal follow-up of the original MIDUS study and included participants aged 49 to 95 at the time of the MRI scan. The MR1 cohort was recruited between 2011 and 2016 to expand representation of middle-aged adults aged 25 to 74 years. Data collection procedures were closely harmonized between the M3 and MR1, allowing the two cohorts to be analyzed within a common methodological framework. Responses to the self-report surveys were obtained 3.8 ± 2.7 years prior to the MRI scan. The final sample consisted of 213 participants (mean age = 56.2 ± 13.6 years; 117 women; 167 White), eligible for complete-case analysis, including 32 African American adults from the MR1 Milwaukee cohort. M3 Milwaukee cohort was not included as the authors had no access to relevant data. The sample size was adequate, as determined by an *a priori* power analysis (**Supporting Information SI-1**). Participant characteristics are summarized in Table 1. Selection-bias analyses indicated that the final analytic sample was younger than those excluded but otherwise comparable across demographic characteristics and baseline health (**SI-2**).

### Key Variables

#### Subjective Well-Being.

To capture the multidimensional nature of subjective well-being, we used the participants’ responses to the following instruments: Psychological Well-Being Scale^7^, Social Well-Being Scale^23^, Life Satisfaction Scale^30^, and Positive and Negative Affect Scale^29^. The Psychological Well-being Scale measures individuals’ positive functioning in terms of sense of purpose, personal growth, autonomy, mastery, self-acceptance, and relationships with others. The Social Well-Being Scale assesses how positively and meaningfully individuals perceive their connection to the broader community across the domains of social integration, acceptance, contribution, actualization, and coherence. The Life Satisfaction Scale measures participants’ evaluations of major life domains, such as work, financial situation, and health. The Positive and Negative Affect Scale assesses the frequency of pleasant and unpleasant emotional experiences during the previous 30 days. Descriptive statistics and example items for each measure and its corresponding subscales are provided in **SI-3**.

#### Covariates.

We controlled for a broad array of variables previously implicated in biological aging and longevity. These include demographics (i.e., age at MRI scan, race, sex, education, and income), baseline health (i.e., body mass index, total number of chronic conditions), lifestyle (i.e., history of drinking and smoking, frequency of weekly exercise and cognitively engaging activities), and broader social contexts that capture the structural aspect of social relationships (i.e., marital/co-residency status, social network size). Educational attainment and annual income were standardized and summed to a composite SES score (see **SI-4** for models analyzing them separately). Raw scores were recoded so that higher values denoted greater magnitude, quantity, or frequency. The list of all focal covariates, their rationale for inclusion, and zero-order correlations with DunedinPACNI are summarized in **SI-5**. We also included the indicator for the MIDUS cohort to account for residual differences between the MIDUS3 and MIDUS Refresher samples that may not be fully captured by the focal covariates.

#### Structural MRI acquisition and preprocessing.

High-resolution T1-weighted anatomical images (1 mm^3^ isotropic) were obtained using a 3T scanner (MR750, GE Healthcare, Waukesha, WI; 8-channel head coil for M3, 32-channel NOVA head coil for MR1) with the following acquisition parameters: repetition time = 8.2 milliseconds (ms), echo time = 3.2 ms, flip angle = 12°, matrix = 256 × 256, field of view = 256 mm, slices = 160, slice thickness = 1 mm, and inversion time = 450 ms. Raw T1-weighted images were processed using FreeSurfer (v.6.0.0)^31^. Cortical surfaces were reconstructed based on the Desikan–Killiany atlas^32^, yielding estimates of thickness, surface area, gray matter volume, and the gray-to-white matter signal intensity ratio for 34 regions per hemisphere. Subcortical and cerebellar volumes were estimated from the automated segmentation function (ASEG). The resulting 315 structural features were subsequently used to compute Dunedin Pace of Aging Calculated from NeuroImaging (DunedinPACNI) for each participant.

#### DunedinPACNI Calculation.

DunedinPACNI is a brain MRI-derived index of multisystem pace of biological aging^28^. The algorithm was developed using data from the Dunedin Multidisciplinary Health and Development Study (*N* = 860) by training an elastic-net regression model to estimate the longitudinal Pace of Aging measure from the structural MRI features described above. The original Pace of Aging phenotype was quantified as within-person changes across 19 biomarkers of cardiovascular, metabolic, renal, immune, dental, and pulmonary functioning, assessed repeatedly at ages 26, 32, 38, and 45 years^33^. The external validity and prognostic relevance of DunedinPACNI have been demonstrated in independent samples of middle-aged and older adults aged approximately 44 to 97 years^34,35^, supporting its application to the age range represented in the MIDUS subsample. Accordingly, the elastic-net coefficients from the original model were applied to the FreeSurfer-derived structural features in the present sample, and the weighted values were summed with the model intercept to generate a DunedinPACNI score for each participant. Higher scores indicate a relatively faster pace of biological aging. Note that DunedinPACNI scores were not separately corrected for chronological age, as it is not a gap-based index prone to systematic estimation bias related to age^36^.

### Statistical Analysis

All analyses were conducted using SPSS (IBM Corp, 2023, version 29) and R (version 4.5.0), with the base significance threshold of α = .05. Exploratory analyses assessing the robustness and specificity of the primary findings are reported in the relevant subsections of the “Results.” This study was not preregistered.

#### Defining Well-Being Dimensions.

Well-being dimensions were defined using confirmatory factor analysis (CFA) based on the hierarchical well-being framework proposed by Gallagher et al (2009)^24^. The analysis was done in two steps. First, using fourteen subscale-level indicators from the Psychological Well-Being Scale, Social Well-Being Scale, Life Satisfaction Scale, and Positive and Negative Affect Scale, we compared the three-factor model with the one- and two-factor models to determine whether the theory-derived tripartite structure was preferable to simpler alternatives in the present sample. The one-factor model specified a single general well-being factor across all indicators. The two-factor model distinguished hedonic well-being from a broader eudaimonic factor spanning the private (intrapersonal) and social (interpersonal) dimensions of well-being. The three-factor model distinguished hedonic well-being, social well-being, and private/eudaimonic well-being (Fig. 1). Note that the original tripartite model used “eudaimonic well-being” to refer to the private or intrapersonal dimension of well-being. Here, we use the term “private/eudaimonic well-being” to avoid confusion with its broader usage in the literature, where eudaimonic well-being may refer to positive functioning across both private and social domains^37^. Relatedly, we did not specify models with more than three well-being dimensions, as developing and validating an expanded framework was beyond the scope of this study. All CFA models were defined using the “*lavaan”* package in R^38^. Next, participant-specific latent factor scores from the best-fitting model were estimated for the primary analyses examining dimensional associations between well-being and DunedinPACNI. See **SI-6** for additional details.

#### Examining associations between well-being dimensions and DunedinPACNI.

Hierarchical multiple regression was used to test whether dimension-specific well-being scores were associated with DunedinPACNI. Separate models were estimated for each CFA-derived well-being dimension, with predictors entered in three sequential blocks, controlling for demographic (Block 1), health/lifestyle (Block 2), and broader social context (Block 3) (Table 2). For the fully adjusted model, we computed 99% bias-corrected and accelerated (BCa) bootstrap confidence intervals (CIs) (10,000 iterations) to assess the robustness of the regression coefficient^39^. The base significance threshold was corrected for the total number of well-being dimensions defined and tested (See Results). Exploratory analyses testing alternative covariate specifications or moderation effects were not corrected for multiple comparisons.

Complementing the hierarchical regression analyses, we conducted variable selection using bootstrapped elastic net regression to test whether the associations between well-being dimensions and DunedinPACNI observed in the regression analyses reemerge without imposing an *a priori* covariate structure and block sequence. This data-driven approach evaluates correlated predictors simultaneously while shrinking weak or redundant coefficients toward zero, allowing their relative predictive information to be assessed within the same model. Accordingly, all well-being dimensions from the best-fitting CFA model, along with covariates, were standardized and entered simultaneously as candidate predictors of DunedinPACNI. Elastic net models were estimated using the “*glmnet*” package in R^40^, with the mixing and regularization parameters selected via 5-fold cross-validation. To assess the stability of variable selection results, the elastic net procedure was repeated across resampled datasets (10,000 iterations), and selection probability was calculated as the proportion of iterations in which each predictor received a nonzero coefficient. Predictors retained with nonzero coefficients were interpreted as variables that carry predictive information for DunedinPACNI in the penalized model, with selection probabilities exceeding 75% indicating high stability across resampling^41^. Detailed procedures are reported in **SI-7**.

## Results

### The 3-factor well-being structure was favored in the model comparison.

The three-factor model (i.e., hedonic, private/eudaimonic, and social well-being) showed acceptable overall fit (CFI = .90, TLI = .89, RMSEA = .07, 90% CI [.06, .08], SRMR = .06). Consistent with Gallagher et al. (2009), its fit indices were uniformly more favorable than those of the one-factor (i.e., general well-being) and two-factor (i.e., hedonic vs. combined eudaimonic well-being) models. Nested model comparisons using the Satorra–Bentler scaled chi-square difference test further confirmed that the three-factor specification fit significantly better than the two-factor model (Δχ^2^ = 31.1, *p* < .001), which in turn fit significantly better than the one-factor model (Δχ^2^ = 34.1, *p* < .001). The resulting well-being dimensions are strongly intercorrelated, as in the original study^24^. Yet these comparisons suggest that modeling them as partially separable factors better represented the observed covariance structure than collapsing them into broader well-being constructs. Participant-specific factor scores were thus estimated from the three-factor model for subsequent analyses, and the type-I error rate for the main analysis was adjusted for three comparisons (α = .0167). Detailed fit indices and factor loadings are provided in **SI-6**.

### Social well-being showed consistent associations with lower DunedinPACNI scores.

Regression diagnostics indicated no evidence of multicollinearity, residual non-normality, or influential cases of concern (**SI-8**). The regression models explained a significant amount of variance in DunedinPACNI scores regardless of the well-being dimensions tested (R^2^ = .30–.40). The addition of health and lifestyle covariates in Block 2 significantly improved model fit (All *P*s = .001), whereas the inclusion of marital status and social network size in Block 3 yielded null effects (All *P*s ≥ .469) (See **SI-9** for model summaries and fit measures).

Importantly, social well-being consistently predicted lower DunedinPACNI across all three hierarchical blocks under the adjusted significance threshold (α = .0167) (Fig. 2a, Table 3). The full test statistics are provided in **SI-10**. This association was not moderated by age, sex, or by MIDUS cohort (All *Ps* > .084). The results also persisted when SES was split into educational attainment and annual income (*p* = .008), and when individual differences in measurement lag between the survey and MRI scan (3.8 ± 2.7 years) were included as an additional covariate (*p* = .004). In contrast, private/eudaimonic well-being and hedonic well-being showed significant and nominally significant negative associations, respectively, only in the baseline demographic block. Both effects attenuated once health and lifestyle covariates were accounted for in Model 2 and remained nonsignificant in the fully adjusted model (Fig. 2b, **SI-10)**. Bootstrapping yielded converging results: 99% BCa bootstrap CIs excluded zero in the fully adjusted model, but only for social well-being (**SI-11**). Across models, older age, higher BMI, and greater chronic disease burden were associated with higher DunedinPACNI scores, whereas higher socioeconomic status was associated with lower DunedinPACNI scores. MIDUS Refresher membership was also inversely linked to the pace of aging, which is expected given the age difference between the cohorts.

### Social well-being was the only well-being dimension retained in variable selection.

The elastic-net analysis complemented the hierarchical regression analysis by evaluating all well-being dimensions simultaneously, allowing correlated predictors to compete for predictive information within a single penalized model. The model retained MIDUS cohort, chronological age, chronic conditions, SES, BMI, and social well-being as nonzero predictors of DunedinPACNI. Bootstrap selection probabilities exceed 90% for SES, chronic conditions, BMI, chronological age at MRI, and cohort membership. Social well-being was also retained with high stability, receiving a nonzero coefficient in 83.3% of bootstrap iterations. Private/eudaimonic and hedonic well-being, by contrast, had low selection probabilities of 14.16% and 4.79%, respectively (Fig. 2c, **SI-7**). These data-driven results suggest that when the correlated well-being dimensions were considered jointly, social well-being more consistently retained predictive information for DunedinPACNI than either hedonic or private/eudaimonic well-being.

### Exploring a modifiable pathway to a slower pace of aging via social well-being.

Given the consistent association between social well-being and DunedinPACNI across different analytic approaches, we conducted an exploratory mediation analysis to examine whether this finer-grained characterization of well-being could reveal a potentially modifiable pathway to slower aging. As a test case, we focused on community volunteering, a prosocial behavior previously implicated in greater subjective well-being^42^, lower mortality risk^43^ and slower epigenetic aging^44^. We tested whether social well-being represented a plausible pathway linking volunteering to a slower pace of aging and whether comparable indirect associations emerged for the other well-being dimensions.

Accordingly, we constructed a mediation model in which total monthly volunteering hours across contexts (i.e., hospital, nursing home, school, other youth-related work, political organization, and other organizations) were summed and entered as the predictor, social well-being as the mediator, and DunedinPACNI as the outcome. Volunteering hours were right-skewed and thus log-transformed. The model was adjusted for the same covariates used in the primary analyses. All mediation analyses were performed using PROCESS macro (version 4.2)^45^.

We found that total monthly hours spent volunteering for various community organizations were associated with lower DunedinPACNI through higher social well-being, as indicated by a significant 99% bootstrapped indirect effect (a×b = − .04, 99% CI [−.100, − .004]) (**SI-12**). The result persisted even with a binarized volunteering-hours variable (**SI-13**). However, this indirect pathway did not emerge when we modeled private/eudaimonic or hedonic well-being as mediators, or when we reversed the predictor and mediator (**SI-14**). To further examine whether this pathway was contingent on the level of construct differentiation, we repeated the analyses using factor scores derived from the one- and two-factor CFA solutions. No significant indirect effects emerged under these broader representations of well-being; the indirect association was detected only when social well-being was distinguished from private/eudaimonic well-being in the three-factor solution (**SI-15**).

## Discussion

Although often measured as a unitary construct in aging research, subjective well-being is multifaceted. This observation raises the question of whether the dimensions of well-being are equally linked to healthy aging, and whether exploring these dimensional associations yields novel insights into identifying targets for preventive interventions. Drawing on the MIDUS study, we modeled separable dimensions of subjective well-being and examined their prospective associations with DunedinPACNI, a novel brain-based pace-of-aging biomarker that extends existing blood-derived aging measures. Lastly, we explored the translational implications of the primary finding using an exploratory mediation model linking community volunteering, well-being dimensions, and DunedinPACNI.

The best-fitting model from confirmatory factor analysis favored three, partially separable dimensions of subjective well-being that align with prior literature: hedonic, private/eudaimonic, and social well-being. Among these dimensions, only social well-being was consistently associated with lower DunedinPACNI scores. This pattern emerged in both the hierarchical regression models and the data-driven variable selection approach.

Social well-being indexes the perceived quality of one’s connection to the broader social world, such as the sense of being integrated into, accepted by, and useful to one’s community^23^. Such appraisals of the social environment engage evolutionarily conserved threat- and safety-detection systems^46^. Perceived social disconnection triggers sustained neuro-inflammatory signaling^47^, whereas perceived integration and contribution are associated with dampened HPA-axis activity^48^. Animal models have shown that these physiological responses can result in accelerated aging measured at multiple levels, including telomere shortening^49^, pathological apoptosis^50^, and functional decline across organ systems, including the brain^51^. It is thus possible that social well-being, closely tied to social experiences that regulate these processes, most sensitively tracks the signatures of pace of aging measured via the brain.

Private/eudaimonic well-being showed a significant association with DunedinPACNI after controlling for demographics. Hedonic well-being remained only nominally significant at baseline. Both relationships attenuated further to null in subsequent regression blocks incorporating BMI and chronic disease burden, each of which independently predicted DunedinPACNI. One interpretation is that hedonic and private/eudaimonic well-being lie partly downstream of these conditions: physical health and material conditions are well-documented determinants of life satisfaction and positive functioning (e.g., purpose and environmental mastery)^52–57^, such that the relevant well-being dimensions explained little variance once these factors were directly modeled. Consistent with this pattern, Kim et al. (2023) found that purpose in life predicted slower epigenetic aging, but this association became nonsignificant after controlling for chronic conditions and health behavior^18^. This potential confounding is also relevant to the broader literature, as many prior studies linking purpose and positive affect to outcomes such as mortality and healthy life expectancy did not comprehensively account for lifestyle factors and baseline health conditions^10,11^. Social well-being, by contrast, may have retained a stronger association because perceptions of one’s functioning within the broader social world are less reducible to personal health status or lifestyle factors^23^.

This interpretation may also explain why structural features of the social environment, such as marital status and social network size, were not significantly correlated with DunedinPACNI. Perceived isolation, loneliness, and quality of social relationships tend to predict morbidity and mortality more strongly, and partly independently of the mere number of social ties or frequency of social contact^58–60^. Our findings are consistent with this distinction between structural and subjective aspects of social connection and extend it to an MRI-derived pace-of-aging marker. This distinction has practical implications, suggesting that interventions could prioritize cultivating a sense of community integration, contribution, and acceptance, rather than simply increasing the quantity of social contact or the size of an individual’s social networks.

One everyday behavior well-suited to cultivating this experience is volunteerism. Volunteering has been linked to indices of healthy aging, such as lower mortality risk, preserved cognitive functioning^61,62^, and slower biological aging measured using DNA-methylation clocks^63^. These associations could be mediated by different dimensions of subjective well-being, as volunteering can elicit positive emotions, strengthen purpose and mastery, and deepen a sense of social integration and contribution^42^. By facilitating coping^64^ and recovery from stress^65^, these psychological resources could limit cumulative physiological strain. In the present study, our exploratory mediation analysis showed that greater monthly volunteering hours were indirectly associated with lower DunedinPACNI through social well-being, but not via hedonic or private/eudaimonic well-being. Likewise, we found no significant indirect associations with subjective well-being defined using the broader one- or two-factor solutions. This pattern has translational relevance, suggesting that the health benefits of volunteering may depend in part on whether it fosters a sense of being needed, integrated, and valued, a pathway that may be obscured when well-being is modeled more broadly. We emphasize, however, that these findings do not imply a causal mechanism. Our study design is observational, and volunteering and well-being were measured concurrently. Establishing whether social well-being shapes the pace of aging beyond statistical mediation will require independent validation via longitudinal and intervention studies, potentially leveraging established programs that promote social participation among older adults (e.g., Experience Corps)^66^.

DunedinPACNI may offer a practical option to facilitate such efforts. The association between well-being and the pace of aging had previously been demonstrated only with blood-derived markers^18^. To our knowledge, this study presents the first evidence that a similar association can also be detected using algorithms trained on structural brain features. We also showed that DunedinPACNI exhibits consistent associations with many established predictors of health and mortality, including SES, BMI, and chronic conditions, further supporting its utility as an aging biomarker. Since DunedinPACNI requires only T1-weighted MRI data, it enables independent validation and data pooling across existing neuroimaging datasets that lack biospecimen measures. This may broaden opportunities to test psychosocial correlates of biological aging and, ultimately, to inform interventions.

### Limitations and Future Directions

Several important limitations warrant discussion. First, although subjective well-being was assessed prior to the MRI scan, we cannot rule out reverse causality: individuals aging more slowly may remain healthier, more active, and more socially engaged, and may consequently appraise their social lives more positively. Longitudinal designs with repeated assessments of well-being and neuroimaging, and intervention studies that experimentally enhance social well-being (e.g., volunteering programs), are needed to test the causal pathway. Second, MIDUS samples with neuroimaging data are predominantly White and may exhibit selective attrition, favoring younger, healthier, and more educated participants^67^. The final analytic sample of the current study was similarly skewed toward middle-aged participants, limiting generalizability to older individuals. Third, DunedinPACNI remains a relatively new biomarker. Its convergent validity with other pace-of-aging indices and the stability of the estimate across demographic strata require further scrutiny^68^, particularly given that different biological aging measures tend to show only moderate correlations with one another^69^. Fourth, although social well-being showed the most consistent association with DunedinPACNI, we did not formally test whether its regression coefficient differed from those of private/eudaimonic or hedonic well-being. The elastic-net analysis partially addressed this issue by comparing the three correlated dimensions simultaneously, but larger sample sizes would enable direct comparison of their unique effects within a multivariate structural equation modeling framework. Finally, latent factor scores necessarily depend on the specific instruments used for measurement. Although our characterization of well-being was grounded in a prior theory that received empirical support^24,70^, replicating the present findings using alternative indicators, potentially in non-MIDUS cohorts, would strengthen the link between social well-being and a slower pace of aging.

## Conclusion

As populations age worldwide, identifying modifiable psychosocial factors that shape the pace of biological aging is a pressing public-health priority. Our findings have two broad implications. First, carefully delineating psychosocial constructs can yield unique insights into pathways that support healthy aging. Second, this dimensional pattern generates testable hypotheses for the design of preventive interventions, pointing to both a candidate mechanism of action (e.g., experiences of community integration and a sense of contribution) and target groups (e.g., individuals with low social well-being despite intact social networks or overall life satisfaction). Replication in larger cohorts, potentially leveraging DunedinPACNI across existing neuroimaging datasets, will help substantiate these findings and determine whether they can inform scalable strategies to promote healthy aging.

## Supplementary Material

Supplementary Files

This is a list of supplementary files associated with this preprint. Click to download.

- SWPACNISupportingInfo.docx

## Figures and Tables

**Figure 1:**
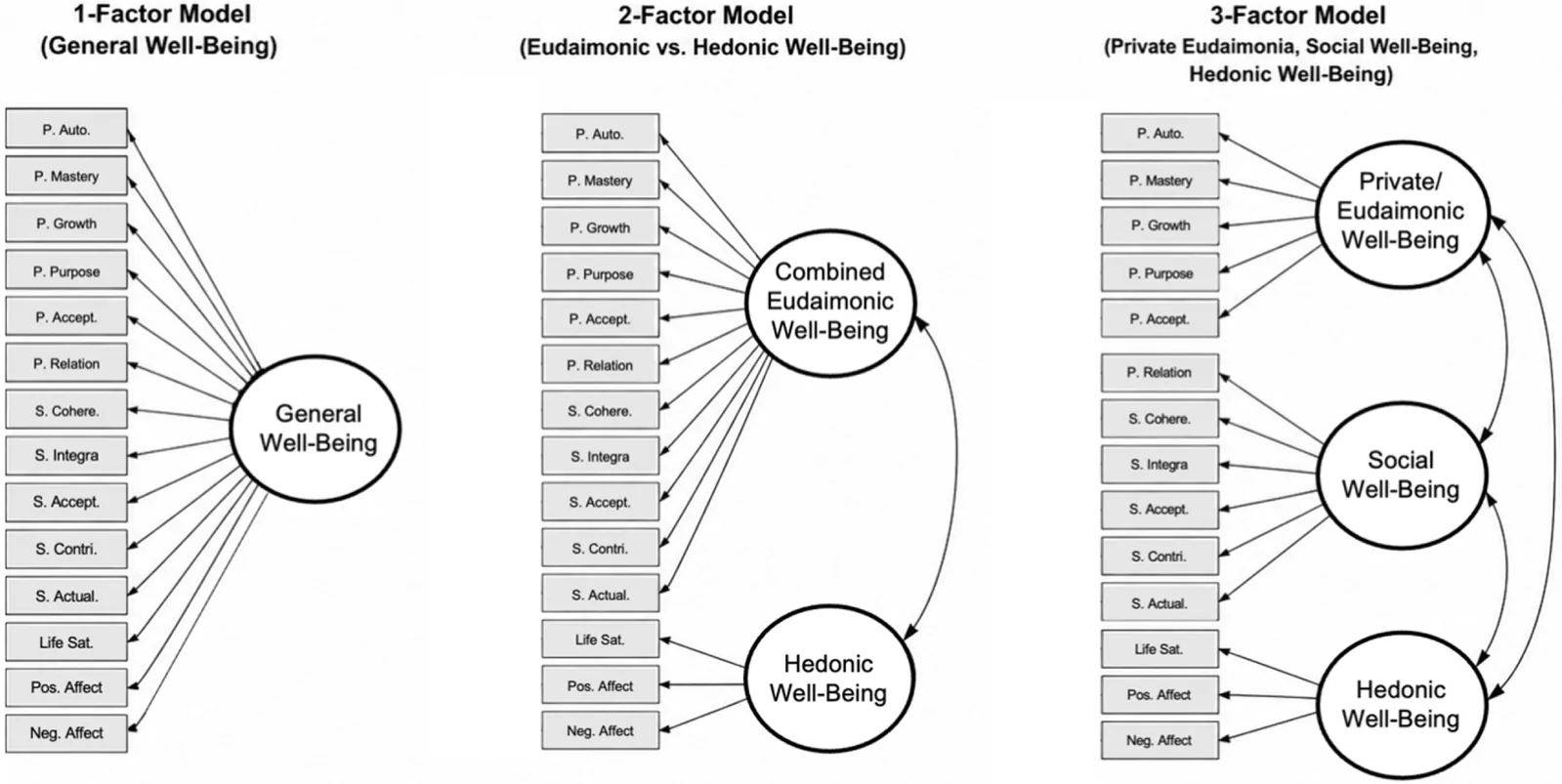
Confirmatory factor analysis models of well-being. All model specifications were based on Gallagher et al (2009). P. Auto. = autonomy; P. Mastery = environmental mastery; P. Growth = personal growth; P. Purpose = purpose in life; P. Accept. = self-acceptance; P. Relation = positive relations with others; S. Cohere. = social coherence; S. Integra. = social integration; S. Accept. = social acceptance; S. Contri. = social contribution; S. Actual. = social actualization; Life Sat. = life satisfaction; Pos. Affect = positive affect; Neg. Affect = negative affect.

**Figure 2:**
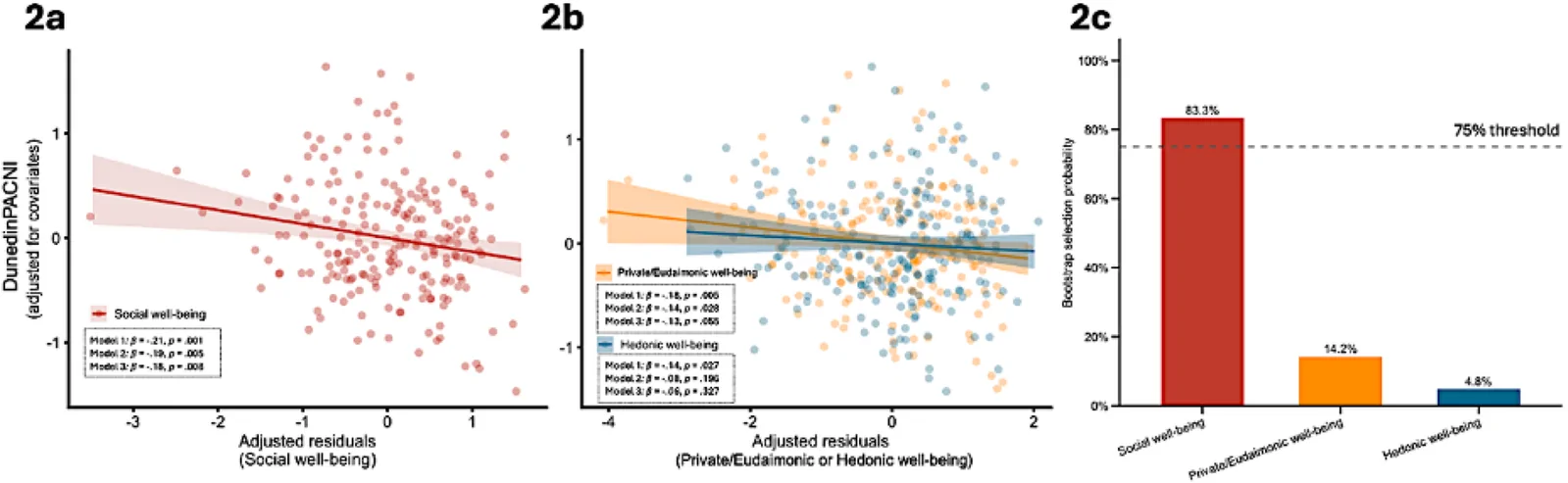
Associations between well-being dimensions and DunedinPACNI estimates. (a) Higher social well-being was associated with lower DunedinPACNI scores across progressively adjusted hierarchical regression models. (b) Associations between other well-being dimensions and DunedinPACNI were no longer significant after adjustment for covariates. Points represent individual participants, solid lines show fitted regression slopes, and shaded bands indicate 95% confidence intervals. Standardized regression coefficients (*β*) are reported. Higher DunedinPACNI scores indicate a faster estimated pace of biological aging. (c) Bootstrap selection probabilities from elastic-net regression for the three well-being dimensions. The dashed line indicates the 75% stability-selection threshold.

**Table 1:** Descriptive statistics of the key variables

Characteristics	Full sample (*N* = 213)
MIDUS Cohort, *n* (%)	
MIDUS 3	102 (47.9%)
MIDUS Refresher 1	111 (52.1%)
Age at MRI (years)	56.2 ± 13.6 (26–95)
Sex, *n* (%)	
Female	117 (54.9%)
Male	96 (45.1%)
Race, *n* (%)	
White	167 (78.4%)
Black	32 (15.0%)
Other	14 (6.6%)
Education, *n* (%)	
Some high school	7 (3.3%)
GED	2 (0.9%)
Graduated from high school	41 (19.2%)
1–2 years of college	30 (14.1%)
3 or more years of college	8 (3.8%)
Graduated from 2-year college / vocational school / associate degree	27 (12.7%)
Bachelor's degree	54 (25.4%)
Some graduate school	3 (1.4%)
Master's degree	32 (15.0%)
PhD, EdD, DDS, JD, LLB, LLD, or other professional degree	9 (4.2%)
Annual Household Income, USD	57,129 ± 55,380 (0–300,000)
Marital Status, *n* (%)	
Married	150 (70.4%)
Not married	63 (29.6%)
Body mass index (BMI)	29.4 ± 5.6 (18.3–46.4)
Chronic conditions (total number)	3.3 ± 2.5 (0–11)
Smoking (Year)	7.4 ± 12.5 (0–45)
Drinking (Year)	7.7 ± 10.5 (0–60)
Exercise frequency (Weekly)	4.8 ± 4.7 (0–19)
Frequency of engaging in cognitively stimulating activities (Weekly)	14.3 ± 4.7 (2–28)
Social Network Size	15.3 ± 11.6 (2–65)
Global Life Satisfaction	7.5 ± 1.3 (3.4–10.0)
Positive Affect	3.4 ± 0.7 (1.5–5.0)
Negative Affect	1.4 ± 0.6 (1.0–4.7)
Psychological Well-Being (Total)	16.9 ± 2.4 (7.2–20.8)
Social Well-Being (Total)	13.4 ± 2.5 (3.4–18.2)
Hours Spent for Community Volunteering (Monthly)	9.9 ± 23.6 (0–216)
DunedinPACNI	0.7 ± 0.7 (−1.4–2.69)

*Note*. Continuous variables are presented as mean ± standard deviation (range), and categorical variables are presented as n (%). BMI = body mass index; GED = General Educational Development; USD = U.S. dollars; DunedinPACNI = Dunedin Pace of Aging Calculated from NeuroImaging, with higher values indicating a faster pace of aging.

**Table 2:** Hierarchical regression analysis block specification

Model #	Predictors
**Model 1**: Baseline Model	race, socioeconomic status (SES)^1^, age, sex, MIDUS cohort, and well-being dimension
**Model 2**: Health/Lifestyle Model	Model 1 + BMI, the total number of chronic conditions, years of drinking, years of smoking, exercise frequency, cognitive activity frequency
**Model 3**: Social Contex Model	Model 2 + marital/co-residency status + social network size^2^

1 Annual income and education level were standardized and summed to a composite SES score (See **SI-4** for analyses examining income and education separately). Social network size reflected the total number of children, close friends, and family/relative with whom participants regularly interact (See **SI-5**).

**Table 3:** Hierarchical regression analysis of the association between social well-being and DunedinPACNI

Predictor variables	Model 1	Model 2	Model 3
Race	−.054	−.037	−.017
**SES**	**−.173** *	**−.190** **	**−.205** **
**Age at MRI scan**	**.554** **	**.488** **	**.497** **
Sex	.030	−.006	−.013
**MIDUS cohort**	**.576** **	**.586** **	**.590** **
**Social Well-Being**	**−.213** **	**−.185** *	**−.176** *
**Body mass index**	–	**.160** *	**.170** *
**Number of chronic conditions**	–	**.175** *	**.164** *
Drinking history	–	.026	.027
Smoking history	–	−.108	−.123^†^
Exercise frequency	–	−.032	−.031
Cognitive activities	–	.046	.045
Marital/co-residency status	–	–	.065
Social network size	–	–	−.025
*R* ^2^	**.322** ***	**.392** ***	**.397** ***
Δ*R*^2^	–	**.07** **	.004

*Note*. *N* = 213. Standardized regression coefficients are shown (*β*). Dependent variable = DunedinPACNI. For regression analyses, the reference categories were Male for sex, White for race, MIDUS Refresher for MIDUS cohort, and Married/co-residing for marital/co-residency status. Significance thresholds for the regression coefficients were Bonferroni-corrected for three comparisons.

† *p* < .05 (nominally significant before Bonferroni-correction);

* *p* < .0167;

** *p* < .0033;

*** *p* < .00033.

## Data Availability

MIDUS data, instruments, protocols, and codebooks are available through the Inter-university Consortium for Political and Social Research (ICPSR) (https://www.icpsr.umich.edu/web/ICPSR/series/203). Restricted-use data from the MIDUS project can be obtained via ICPSR, and raw structural imaging data are available from the MIDUS Neuroscience Repository (https://midus.wisc.edu/midus-neuroscience-repository/) upon application and approval. Detailed descriptions of the variables used are fully provided in the manuscript. DunedinPACNI is accessible through: https://github.com/etw11/DunedinPACNI. R and SPSS Scripts used for analyses can be obtained upon request.
